# Practical Medicine

**Published:** 1858-11

**Authors:** 


### PRACTICAL MEDICINE.

1. AN ACCOUNT OF SOME CASES OF SCARLATINA ANGINOSA, SUCCESSFULLY TREATED BY HOT PACKING, INDUCING A COPIOUS AND PROLONGED DIAPHORESIS. By *Mr. F. A. Bulley, F.R.C.S.*, Senior Surgeon to the Royal Berkshire Hospital, Reading.—CASE I. Friday, April 30, 1858. I was requested to visit Jane A., a housemaid, aged twenty, who had been suffering for several days previously from fever; there was dry burning heat of the skin, intense and constant thirst, quick pulse, aching pains in the loins and limbs, with an anxious and somewhat confused appearance of the countenance, and all the other well-marked indications of considerable febrile disturbance.

These symptoms had been preceded by coldness of the surface and general chilliness of the body, with a scanty secretion of urine. On the Wednesday previous to my visit, she had begun to feel some uneasiness about the throat, and difficulty in swallowing, which increased so rapidly that, on the next day, Thursday, she found herself unable to swallow without great pain. There was considerable external swelling of the cervical glands; internally, the tonsils were much enlarged, acutely inflamed, and extensively ulcerated on their surface, the inflammation and ulceration extending, as well as I could see, into the pharynx. There was a constant discharge of mucus, somewhat fetid, from the nares.

To day, Friday, April 30, all the symptoms enumerated have increased in intensity; the skin is burning hot. Pulse 125; eyes suffused; great and constant headache.

Saturday, March 1.—The characteristic scarlet eruption has appeared over the neck and forepart of the chest and abdomen; but without any relief to the febrile symptoms, which continue unabated. To relieve the throat, she was ordered a gargle of vinegar and tincture of myrrh, hot vinegar and water fomentations externally, and to inhale the fumes of hot vinegar and water diffused about the room.

Sunday, 2d.—Throat somewhat easier, swallows a little better. The general febrile symptoms continuing unabated, and the eruption not appearing to display itself sufficiently in the extremities, she was ordered to be wrapped up in the hot packing, according to the method more particularly described in the next case.*

A copious diaphoresis having been shortly induced by these means, she was allowed to remain in it for five or six hours, when the wrappings were gradually and carefully removed. At the end of this time her pulse had fallen from 125 to below 100, her headache had almost disappeared, a gentle moisture bedewed the skin, and, after expressing herself as greatly relieved by the sweating, she fell into an easy undisturbed sleep, and slept for several hours.

The burning heat of the skin has altogether left her. She has little or no thirst; skin moist and perspiring.

She feels somewhat weak from the effects of the packing. To take strong beef-tea constantly during the day; she was ordered, for common drink, to take two teaspoonfuls of vinegar in a tumbler full of cold spring water, for the purpose of keeping up the diaphoresis, and an ordinary saline draught at intervals during the day.

The future progress of this case may be thus briefly stated. The inflammation and swelling of the throat gradually subsided under the use of a mixture of honey and water with tincture of myrrh inhaled from Dr. Harwood's inhaler; the sloughs subsequently became detached from the tonsils, exposing a superficially ulcerated surface underneath. The burning heat of the surface never returned, a gentle perspiration remained constantly upon the body; the pain in the loins left her after two or three days, and the secretion of urine returned: on the fifth day after the application of the hot packing, her appetite for solid food was returning, and on the sixth day, or about the tenth from the first accession of the febrile symptoms, it had completely returned, and she might in every respect be said to be quite convalescent from the disease. It may be as well to mention that no desquamation of the cuticle ensued on convalescence.

---

* In this case, however, the patient's body was not immersed in tepid water; but thick pads of folded household flannel, wrung out of very hot water, were placed upon the chest and abdomen previous to the packing.

CASE. II. July 4, 1858.—I was requested to visit Ellen K., a very delicate-looking child, aged seven and a half years, who had been attacked with scarlatina.

The characteristic eruption had appeared the day before I saw her; it was more particularly observable over the trunk of the body, but very little displayed upon the extremities, except in a few isolated patches over the wrists and ankles.

It had been preceded by dry burning heat of the skin, which still continued, with constant and severe headache. Great thirst; pulse 130 in the minute; eyes suffused; occasionally severe paroxysms of coughing, and some difficulty in her breathing; great anxiety of countenance.

On examining the throat I found the tonsils very much enlarged, especially the left, and for the most part covered with a thick light-colored tenacious slough; the portions unoccupied by the slough were of a bright-red color, apparently in a state of intense inflammation. She had the greatest difficulty in swallowing anything at all solid. Externally the cervical glands were swelled, and extremely tender to the touch.

As I had frequently had occasion to observe the beneficial effects of a copious and sustained diaphoresis in the treatment of the various forms of scarlatina, she was immediately submitted to this mode of treatment, which was carried out in the following manner:—

Her body having been immersed in tepid water for about half a minute, at 75° Fahrenheit, she was laid, dripping, upon a coarse sheet previously a little warmed, which was then closely wrapped round her, more particularly about the neck, to confine the heat generated by the process.

This coarse sheet had been previously laid upon two blankets in the bed, and now these blankets were brought over in the same manner as the sheet, thus closely enveloping the body. Over these primary envelopes, other blankets to the number of eight or ten were laid until she could no longer bear the superincumbent weight. In about twenty minutes after the application, perspiration began to appear upon the face, and on examination shortly afterwards, it was found that the whole body was bathed in a copious diaphoresis, in which she was compelled to remain for about five hours, when the envelopes were carefully and gradually removed; the sheet which had formed the immediate envelope to the body was found to be completely saturated by the perspiration of the patient. When the patient had been entirely removed from the wrappings, and placed in a dry warm bed, she expressed herself as feeling much better, her pulse was diminished to 90 in the minute, her headache and thirst had become greatly relieved, and her body continued in a comfortable moisture throughout the remainder of the night.

On my visit next morning, I found that the same gentle perspiration had continued, her breathing had become much more easy, and she had scarcely any thirst; the eruption was rather more vivid than before the packing; her throat was still swelled externally, but certainly not so much so as before the application; internally, the inflammation was not so vivid, but the sloughs were still as adherent as before; for this she was ordered to take lemon-water ice in small quantities during the day, with, occasionally, small pieces of rough lake ice, which appeared to afford her great relief. For ordinary drink, she was ordered to take a weak mixture of brown vinegar and spring water, as a simple diaphoretic. To have strong beef-tea, cold, frequently during the day, and to take a saline draught, with small doses of the chlorate of potash, two or three times daily.

Flannel cloths steeped in hot vinegar and water, to be kept constantly applied to the outside of the neck and throat.

It would be tedious to follow the progress of this case to its successful termination; suffice it to say, that from the period of the discontinuance of the sudatory process which I have described, the little patient never experienced any return of the dry burning heat which had previously affected her; a gentle moisture constantly suffused her skin, her thirst gradually ceased, and her appetite for nourishing food returned; in the mean time, the external swelling of the throat subsided; internally, the sloughs became separated, and exposed a healthy surface underneath, the tonsils gradually assuming their natural healthy appearance.

On the fourth day she took a mutton chop, with a quarter of a pint of bitter ale, with a relish; on the sixth day she was well enough to sit up; and on the eighth day from the first accession of the symptoms, as she appeared to have in every respect completely recovered from her disease, with the exception of the debility to be expected after such a severe attack, she was removed into the country, where she has completely recovered her health and strength.

No desquamation of the cuticle had followed the disappearance of the scarlet rash.

Case III.—I have not myself had an opportunity of testing the efficacy of this mode of treatment in the anasarca which sometimes follows scarlatina; but my friend, Mr. Winter Dryland, the resident-surgeon of the Reading Dispensary, who has recently had a considerable number of cases of scarlatina under his care, has given me the particulars of a case of this kind, in which its effects were so immediate and striking, that he thought it right to inform me of the facts, and has kindly permitted me to publish them.

A little child, aged six years, had apparently recovered from a somewhat severe attack of scarlatina, when, about a fortnight after its apparent convalescence, it was suddenly attacked with symptoms of anasarca of the limbs; great prostration soon followed; the secretion of urine became diminished; a difficulty of breathing ensued, indicating, at least, the commencement of effusion into the cavity of the thorax, the dyspnœa gradually but perceptibly increasing. In this emergency, Mr. Dryland thought he would resort to the treatment of hot packing, which was accordingly carried out in the manner described in the foregoing cases. By this means a copious diaphoresis was shortly induced, in which the little patient was kept for five or six hours, during which time, owing to its previous exhaustion, and the additional debility produced by the process, it was found necessary to supply it frequently with small quantities of warm brandy and water to prevent its sinking. At the end of this period, Mr. Dryland, who had carefully watched the patient throughout the whole of the process, observed that the great difficulty of breathing had partially disappeared, and, in some respects, the anasarca of the extremities; to use his own words, "there was an obvious diminution of the swelling of the extremities on the removal of the packings," and from this moment, under the judicious use of chalybeate remedies, the child gradually recovered from the attack, and was shortly afterwards quite convalescent.

*Remarks.*—It would be difficult to explain exactly the *ratio medendi* of the diaphoretic means employed in the foregoing cases. In the generality of instances, it would seem to act by subduing the inflammation of the skin which attends this disease, which, like the inflammation of all other secreting organs and tissues, prevents its performing properly, or even at all, its healthy secretory functions. This suspension or arrest of its natural functions causes a greater quantity of blood to be thrown upon other organs of secretion, especially the kidneys and tonsils, and the glandular apparatus of the intestines, which thus, in turn, become congested and inflamed, and after a time also lose their secretory power, ultimately becoming disorganized from the continuance of the congestion. Thus, in the end, in the worst case of this disease, all the excretory functions become suspended, and the patient dies, to all intents and purposes poisoned by his own blood.

It was to modify, and, if possible, to remove this primary inflammation of the skin, and to prevent its secondary operation on the other organs of the body, that I was induced to adopt the treatment I have described, with what success may be partially judged of by the details of the foregoing cases, selected from a large number of others treated in the same manner during the last fifteen years; and I have the satisfaction to remark that, during this period, I have never lost a single patient from this disease, however malignant the case might appear to be, which has been treated in this manner.

Something may also be due to the eliminatory powers of the prolonged sweating process employed, and from the manner in which I have found it act in other kinds of fever, in which I have extensively used it. I believe it possesses the faculty of eliminating whatever poison there may be in the disease from the system, at least to an equal degree in scarlatina. The rapid manner in which the febrile symptoms have subsided under its use, and the absence of their return in many cases, have led me to this conclusion, and I have no doubt that its good effects may partly be attributable to this power, combined with its more immediate action upon the skin, as I have described.

The medicinal treatment employed in these cases was almost of a negative character; ice, in small pieces, frequently swallowed, seemed to afford great relief to the throat. The beneficial effects of ice in these cases have long been known to the profession in assuaging tonsillary inflammation; but children of tender age frequently object to it in a rough state, and I have therefore generally substituted for it either lemon-water, or strawberry-ice, from the confectioners, which I have generally found the little patients take with pleasure, and even avidity.

A weak diluted solution of vinegar and water was administered as a common antiseptic drink, sweetened and rendered palatable by a small quantity of sugar. Vinegar taken in this way appears to act gently upon the skin, and keeps up a modified continuance of the diaphoresis produced by the packing. My friend, Mr. Baker Brown, has, I believe, lately published a pamphlet on the use of acetic acid in scarlatina, attributing some very remarkable effects to its medicinal use; but I have not yet had an opportunity of reading it, and, therefore, cannot say to what particular mode of action he refers its remedial superiority. I can only say, that I have used it for a long time dietetically in the manner I have mentioned, and have every reason to feel satisfied with its good effects.—*Medical Times and Gazette*, September 11, 1858.

2. TREATMENT AND CLINICAL HISTORY OF ASTHMA. By *Hyde Salter, M.D., F.R.S.*—In that mild but annoying form of asthma that accompanies the other symptoms of hay-fever, and is known as hay-asthma, tobacco pushed *ad nauseam* gives more relief than any other remedy. In a relative of mine who is very much afflicted with this troublesome complaint, tobacco-smoking is the only thing that gives any relief. During the hay season, and all the early hot summer weather, he suffers (besides the sneezing and running at the eyes, and tumid burning of the nose and throat, characteristic of hay-fever) from paroxysms of a wheezing dyspnœa of the true asthmatic type, coming on exclusively at night, so as almost to deprive him of sleep. During the rest of the year he never smokes, as it is disagreeable to him, and in other respects prejudicial; but during this season he is quite dependent on his cigar for any degree of comfort or alleviation of his symptoms. The following graphic account of the relief he finds from tobacco I cannot do better than give in his own words :—

"There is no remedy during a paroxysm of hay-asthma that has anything like the effect of smoking tobacco; and though this is especially the case in the latter stage of the attack, when the asthmatic element of the phenomena is most developed, still in the earlier stage, when the lachrymation, sneezing, and faucial irritation are most distressing, tobacco-smoke has, in my case, a very marked influence in soothing and diminishing these symptoms.

"No doubt any of those medicines which Dr. Pereira has called '*cardiaco-vascular depressants*,' would produce a somewhat similar result; but none is of so easy application, or can be used so readily or pleasantly as tobacco. During the hay-asthma season—*i. e.*, in my case, from about the 15th of May to the 10th or 12th of July, I regularly smoke a cigar the last thing before going to bed, or perhaps more frequently after I am in bed. The effect is, that (excepting during the last fortnight in June, when I never get a night's rest) the sedative influence of the tobacco prevents the occurrence of any asthmatic spasm. If, during this period, I omit my cigar, I seldom sleep beyond four o'clock; usually three o'clock finds me awake, hopelessly, though generally only slightly, asthmatic for the rest of the night; till, indeed, about nine o'clock, when almost always the asthma completely leaves me. This night-cigar is taken as a preventive. But tobacco will cure the asthmatic spasm when it is fairly on; only it requires a larger dose of the poison, and in a stronger form. The sedative influence of the cigar will usually insure me a fair night's rest; but the powerful depression of strong shag-tobacco is necessary to cut short the spasm when it is established. Even when I do smoke my night-cigar, I not unfrequently have to get up about three or four o'clock in the morning and smoke; and during the last fortnight in June, this happens almost nightly.

"Distressing as are the sensations of collapse from tobacco-poisoning, they are an unspeakable relief when contrasted with the impending suffocation of asthma. I shall never forget an attack which I once had, and the joy with which I hailed the approach of collapse from tobacco-poisoning. It was late in July, many years ago. I had gone into Dorsetshire to stay with a relative in a country house. Immediately surrounding the house were grazing-fields—not hay-fields—and they had not been mown. In this field was a grass—*Nardus stricta*, I think—still blooming luxuriantly; for it is a grass which cattle will not eat; and thus, though past the usual time for hay-asthma, I was accidentally surrounded by its most potent cause—grass in flower. The night came, and I had not been an hour in bed, when I was attacked with the most violent asthma I ever experienced. There were no cigars in the house; but one of the servants had some rank shag-tobacco. I smoked one pipe, then another; and as my face blanched, and my pulse failed, and the cold sweat stood on my forehead, miserable as were the sensations of collapse, they were Paradise to the agonies of suffocation. I shall never forget those moments of relief.

"The story of this attack of asthma, by-the-way, is a very instructive one; and I may just add it here, in brief:—I left my friend's house the day after this paroxysm, and went to the sea-side, where I was as usual perfectly well. Two days after, I received a letter from him asking me to return, as he had had the grass in flower about his house cut down. I did so, and remained with him a fortnight, sleeping every night as placidly as an infant. The *Nardus stricta* had given me the asthma: the scythe had cured it.

"To return to the tobacco. A hay-asthmatic should never smoke tobacco but for his malady. Smoking should never be to him a habit or a meal, for it then ceases to be a medicine. Indeed, to him it should be a deadly drug, for it is by poisoning that it cures."

Not long ago, I was conversing on the subject of his malady with a surgeon of some distinction in this city, who is grievously victimized with hay-asthma, and on asking him what he found do him any good, he replied, "Tobacco; tobacco is the only thing; nothing does me any good but smoking;" and he went on to tell me, that whenever he finds his asthma very bad, and that he shall get no sleep without it, he immediately resorts to a cigar. But the smoking does him no good unless it produces a condition of collapse; the mere sedative effect of it is of no use to him whatever; and having lost, from the habit of smoking, an easy susceptibility to tobacco influence, he adopts the following device to secure its more potent effect: he fills his mouth with tobacco-smoke, and then, instead of breathing it out again at once, as is usual in smoking, retains it in his mouth for several seconds, perhaps a quarter of a minute, then takes another mouthful, and so on. In this way, he finds that the tobacco is more rapidly absorbed by the mucous membrane of the oral cavity, and that a state of collapse is speedily induced. The moment the faintness and sickness comes over him, the asthma ceases, he turns into bed, and has a good night.

The effect of antimony nearly resembles that of tobacco, and it acts in the same way, but the nausea and collapse it produces are long and tedious.

Of the three drugs, I should say ipecacuanha is the most manageable, and entails the least suffering; tobacco the most speedy and effectual.

There are one or two practical points on which I would wish to add a few words.

Remedies of this kind, given with the view of cutting short the paroxysm, should be given as early as possible; and for two reasons: first, because it is much easier to break through the asthmatic condition when it is but just established, while the longer it is allowed to go on, the more inveterate and uncontrollable it becomes, and the more difficult it is to arrest it; indeed, its giving way at all may depend on the earliness with which the remedy is applied. I have known treatment powerless after the dyspnœa has continued for some hours, which never failed if administered as soon as it declared itself. Just at starting, in the earliest stages of the paroxysm, a very slight thing will determine its advance or retreat, and in proportion as it advances and deepens, in just such proportion do remedies become inoperative. The other reason is, that if the spasm *does* yield in spite of having been some time established, the recovery is not so complete as if the remedy had been applied immediately on its appearance. The longer the bronchial stricture lasts, the greater is the arrears of breathing, and the resulting pulmonary congestion; and if this goes on unchecked and increasing for many hours, the disturbance of the vascular balance becomes so great, the capillaries of the lungs so loaded, that it is a long time, many hours, or perhaps even days, before that balance is restored, and the vessels recover their normal condition; and although the bronchial spasm may completely give way, there remains a certain amount of shortness of breath and an incapacity for exertion, and it is not until an abundant expectoration of mucus has taken place, by the pouring out of which the loaded vessels have relieved themselves, that the chest becomes clear and the breathing free. In asthma at once cut short, there is no such accumulated congestion—no mucous exudation, and when the bronchial spasm ceases, all dyspnœa vanishes. If on first awaking with the sensation of asthma, the asthmatic nauseates himself with tobacco, or smokes his nitre-paper, or keeps himself in a standing posture, or in any other way cuts short the paroxysm, he will be throughout the succeeding day exactly the same, with the exception of the sleep he has lost, as if nothing had occurred; but if he suffers the fight between asthma and sleep to go on long, and then on the first remission of the dyspnœa lies back and goes to sleep, he will protract the asthmatic state, deepen the consequent pulmonary arrears, and not only postpone his recovery for many hours, but make it then slow and imperfect. I know an asthmatic who now never loses a day by his disease, in consequence of the promptitude with which he meets its first appearance in the early morning, but who formerly, from continuing to lie in bed, and try to get sleep after the asthma had begun, protracted his sufferings through the day. He is attacked as often as ever, and at the same time—about three or four o'clock in the morning, but the moment he finds his asthma on him, he takes measures to keep himself wide awake—stands leaning against a piece of furniture, and, if necessary, induces tobacco collapse, so that instead of a day's asthma he has half an hour's, and, as far as all the engagements of life go, has ceased to be an asthmatic.

It is a difficult thing for the asthmatic, I know, overwhelmed with sleep as he is, and generally with a peculiarly heavy drowsiness upon him, to leave his bed, or light and smoke his pipe; but he *must* do it; he must rouse himself fairly up, and adopt at once those remedies that in his particular case are most efficacious. In fact, the treatment of the asthmatic paroxysm should be so prompt as to be almost rather preventive than curative: in the treatment of no disease is the injunction "*obsta principiis*" of more vital importance.

One is sometimes asked—Which is the best form of tobacco to use, a cigar or a pipe? I think a pipe has the advantage of more certain strength; cigars vary so much, even the same sort. The tobacco that I generally employ is bird's-eye, as being a mild tobacco, and one by which you run little risk of inducing alarming collapse. *Shag*, or any other of the strong tobaccoes, should not be used by the uninitiated, as the collapse they produce is apt to become protracted and unmanageable. For ladies and young children, a few whiffs of a mild cigarette are quite sufficient.

Of ipecacuanha, I think the powder is better than the wine. I never give a very small dose, it is uncertain and teasing. I would say, always give such a dose as will be certain to secure its own prompt rejection. I never give less than twenty grains, however young the patient may be: it never does harm.

But ipecacuanha is a nauseous thing; and especially to those who have frequently taken it as an emetic, it becomes almost intolerable. I have lately discovered that it may be taken very pleasantly and very efficaciously in the form of some strong ipecacuanha lozenges made by Messrs. Corbyn, of 300 Holborn. They are about four times the strength of ordinary ipecacuanha lozenges; three of them will produce prompt vomiting. They are very convenient, too, for keeping up a slight nausea; and for children they are invaluable. If vomiting is desired, they should be bitten and ground up in the mouth, and swallowed at once.

There is one circumstance that greatly detracts from the utility of tobacco in the treatment of asthma; that practically, indeed, almost destroys it. Our adult male population have so habituated themselves to its use, that they have lost the susceptibility to its full influence, and cannot induce complete collapse by any amount of smoking. Now adult males constitute by far the majority of the subjects of spasmodic asthma; and thus the habit of smoking has rendered powerless, in a large number of cases, what I think may, without any qualification, be called its most potent remedy.

To the practical I need not apologize for these trifling hints, of which I know they will recognize the value.—*London Lancet*, September 18, 1858.

